# Resilience, Life Satisfaction, and Well-Being in Portuguese Adolescents: A Cross-Sectional Study

**DOI:** 10.3390/bs15121743

**Published:** 2025-12-16

**Authors:** Luís Loureiro, Cândida Loureiro, José Santos

**Affiliations:** Escola Superior de Enfermagem de Coimbra, Uniersity of Coimbra, Av. Bissaya Barreto s/n, Celas, 3004-011 Coimbra, Portugal; candida@esenfc.pt (C.L.); jcsantos@esenfc.pt (J.S.)

**Keywords:** resilience, life satisfaction, mental well-being, adolescence

## Abstract

Background: Adolescence is a crucial stage of development marked by significant biological, social, and emotional changes that influence mental health and well-being. This study aims to (a) assess the relationship between resilience, life satisfaction, and mental well-being in adolescents aged between 10 and 18 years who are attending 5th to 12th grade in the central region of mainland Portugal, and (b) assess the quality of fit of a structural mediation model in which life satisfaction mediates the relationship between resilience and mental well-being. Methods: A total of 589 adolescents participated in the study and responded to the *Escala de Avaliação do Eu Resiliente*, the Multidimensional Life Satisfaction Scale for Adolescents, and the Mental Health Continuum–Short Form. Descriptive analyses, ANOVA, canonical correlation analysis, and structural equation modeling with mediation were carried out. Results: A high canonical correlation (*r* = 0.873, *p* < 0.001) was found between resilience and well-being/life satisfaction. Mediation analysis revealed that life satisfaction partially mediates the relationship between resilience and positive mental health (*r*^2^ = 0.34), indicating direct and indirect effects. Conclusions: These results suggest that more resilient adolescents tend to report higher levels of life satisfaction and well-being, which reinforces the importance of promoting resilience and life satisfaction as protective factors for mental health during adolescence.

## 1. Introduction

Adolescence is a critical stage of human development characterized by multiple biopsychosocial changes. While often viewed as a period of significant growth, it simultaneously presents considerable challenges that can engender conflicts and contradictions, consequently impacting adolescents’ mental health (MH) and well-being (WB) (World Health Organization, 2025).

This phase is crucial for the development of fundamental psychological processes that contribute to the formation of one’s identity, cognitive frameworks, and overall perception of the world. Key developmental tasks established during adolescence include cultivating a sense of competence, achieving autonomy, establishing meaning in life, safeguarding personal integrity, and successfully managing and regulating emotional instability (Maslak, 2022).

Moreover, the need for group affiliation and peer acceptance acquires particular salience during this stage, representing a fundamental social determinant of mental well-being (WB). However, although most adolescents have good MH, feel motivated to face challenges, and perform well at school (Arslan, 2021; Leibovich et al., 2018), others may become vulnerable to psychological distress or even mental illness (World Health Organization, 2025) due to multiple physical, emotional, and social changes, as well as contextual difficulties. As previously mentioned, adolescence is a time of significant change. While these transformations can be exciting, they also come with new relationships, friendships, and responsibilities, which can create constant pressure and be highly stressful. This pressure can exacerbate anxiety and internal conflicts, making adolescents feel overwhelmed and insecure (Núñez-Regueiro & Núñez-Regueiro, 2021).

At the same time, exposure to contextual factors such as violence, alcohol and other substance use, risky sexual behaviors, and excessive social media use can increase the likelihood of behaviors that contribute to the development of mental disorders and negatively impact adolescents’ WB (Haidt, 2024; World Health Organization, 2018a, 2018b).

In this article, we work with three concepts: resilience, life satisfaction and mental well-being. Resilience can be understood as a dynamic process of positive adaptation in the face of adversity and stress, involving internal resources and coping strategies that enable the individual to deal effectively with challenges. Life satisfaction, in turn, refers to the global cognitive evaluation an individual makes of their quality of life and the degree of congruence between expectations and outcomes. Mental well-being comprises emotional, cognitive, and social components that reflect psychological balance, adaptive functioning, and positive emotions.

There is empirical evidence showing positive associations among these three components, indicating that resilient adolescents tend to interpret challenges in a more adaptive way, which contributes to more positive evaluations of their own lives and, consequently, to higher levels of psychological well-being.

In this context, some worrying statistics emerge. The World Health Organization (2022) estimates that around 20% of children and adolescents experience at least one mental disorder before reaching the age of 18, with around half of these conditions beginning at the age of 14. However, most cases go undetected or untreated, meaning that a substantial majority will most likely continue into adulthood.

More specifically, data from the Health Behavior in School-Aged Children (HBSC) study (Gaspar et al., 2022) reveal that, although the vast majority of Portuguese adolescents consider themselves happy (72.3%), there has been a decrease in life satisfaction (from an average of 7.68 in 2018 to 7.50 in 2022), as well as a decrease in perceived happiness (from 18.3% of participants feeling unhappy in 2018 to 27.7% in 2022). There has also been an increase in physical and psychological symptoms, such as back pain, headaches, nervousness, irritability, sadness, and fear.

In this regard, the amount of time that Portuguese adolescents spend in front of screens is worrying, given the increasing use of cell phones for online communication (62.4%) and the internet as a means of escaping negative feelings (47.6%). In the aforementioned study, 32.2% of adolescents said they had tried and failed to spend less time on social media (Gaspar et al., 2022).

However, although adolescents are exposed to various risk factors that could lead to MH problems, there is no substantial evidence that these factors alone cause such problems. Some authors suggest that this is due to adolescents developing resilience associated with protective factors. Therefore, it is essential to promote a balance between risk and protective factors. The following protective factors stand out: family environment, family support, social support networks, the ability to ask for and receive help, and other factors that promote family health (Santos et al., 2021).

Evidence shows that resilience is a key factor in positive adolescent development, acting as a protective mechanism in the face of adversity. Rather than being an individual characteristic, resilience is the result of a dynamic process reflecting the interaction between personal, family, and contextual factors, which enables adolescents to manage everyday challenges effectively (Mesman et al., 2021).

Several recent studies show that high levels of resilience are associated with greater mental WB and life satisfaction, which contribute to greater emotional balance, a stronger sense of control over life, and a reinforced sense of self-efficacy. The evidence supports the notion that adolescents with adequate resilience tend to be more optimistic and have better social skills and a more positive self-perception, promoting healthy growth and WB (Usán et al., 2022; Marquez et al., 2023; Carvalho et al., 2024; Zhu et al., 2025).

Despite the substantial evidence correlating resilience, life satisfaction, and mental well-being in adolescents, the explanatory framework of their structural and processual interdependence remains insufficiently explored. Therefore, this study aims to: (a) assess the relationship between resilience, life satisfaction, and mental WB in adolescents aged between 10 and 18 years attending 5th grade to 12th grade in the central region of mainland Portugal; and (b) assess the quality of fit of a structural mediation model in which life satisfaction mediates the relationship between resilience and mental WB.

We tested the following hypothesis: a structural mediation model is anticipated, wherein Life Satisfaction functions as the mediator between Resilience and Mental Well-being (WB), demonstrating an adequate fit to the empirical data.

## 2. Materials and Methods

### 2.1. Participants

The sample consisted of 589 adolescents, 278 (47.2%) of whom were boys and 311 (52.8%) were girls. In terms of educational attainment, 140 (23.8%) were in 5th and 6th grades, 278 (47.2%) were in 7th to 9th grades, and 171 (29%) were in 10th to 12th grades. Their mean age was 13.88 years (*SD* = 2.28).

When comparisons were made between sociodemographic variables to assess the homogeneity or heterogeneity of the sample, all differences except for age, which varies according to educational attainment (F = 1222.44; *p* < 0.001), were not statistically significant.

### 2.2. Procedure

The researchers collected data in person in the classroom. In addition to sociodemographic questions, the survey included three scales. On average, it took 25 min to complete the survey.

Participation in the study was voluntary, and participants could withdraw at any time without facing any consequences. Due to the characteristics of the sample, a consent form was provided for parents or guardians to sign. Adolescents aged 18 signed their own consent form.

Both the study and the survey were approved by the Directorate-General for Education through the Monitoring of School Surveys (Opinion no. 0224900009) and the Ethics Committee of the Health Sciences Research Unit: Nursing (P-736; P-738; P-739; P-740; P-741).

### 2.3. Measures

The survey included the following instruments:

#### 2.3.1. Mental Health Continuum–Short Form

The MHC-SF (Keyes et al., 2008) includes 14 items rated on a Likert-type scale from 0 (never) to 5 (every day). It assesses positive MH across three dimensions: emotional WB, social WB, and psychological WB (Loureiro et al., 2025). Emotional WB includes three items: 1. happy; 2. interested in life; and 3. satisfied with your life. Social WB includes five items: 4. that you have something important to contribute to society; 5. that you belong to a community (like a social group, your school, or your neighborhood); 6. that our society is a good place or is becoming a better place for all people; 7. that people are basically good; and 8. that the way our society works makes sense to you. Finally, psychological WB includes six items: 9. that you like most parts of your personality; 10. that you are good at managing the responsibilities of your daily life; 11. that you have warm and trusting relationships with others; 12. that you have experiences that challenged you to grow and become a better person; 13. that you are confident to think or express your own ideas and opinions; and 14. that your life has a sense of direction or meaning to it.

#### 2.3.2. Escala de Avaliação do Eu Resiliente (Resilient Self-Assessment Scale, RSAS)

The RSAS (Jardim & Pereira, 2006) includes 14 items rated on a Likert-type scale from 1 (never) to 5 (almost always). These items assess resilience across the following four dimensions:

(a) External supports or “I have” (four items that analyze external resources, e.g., “I have people around me I trust and who love me, no matter what”; α total scale = 0.82);
(b) Inner strengths or “I am” (three items that assess internal personal strengths, e.g., “I am a person people can like and love”; α total scale = 0.73);
(c) Social skills or “I can” (five items that encompass the interpersonal skills that allow individuals to discuss their concerns and find solutions to their problems, e.g., “I can talk to others about things that frighten me or bother me”; α total scale = 0.86); and
(d) Willingness to act or “I am willing” (two items that assess the individual’s level of responsibility and self-confidence; α total scale = 0.69).

The scale had high internal consistency (α total scale = 0.91).

#### 2.3.3. Multidimensional Life Satisfaction Scale for Adolescents (MLSSA)

The MLSSA (Segabinazi et al., 2010) consists of 52 items rated on a Likert-type scale from 1 (*not at all*) to 5 (*very much*). These items are divided into the following seven components:

(a) Family: It measures satisfaction with the family environment and includes ten items that describe a healthy, harmonious, affectionate family environment with satisfying relationships (α total scale = 0.89).
(b) Self: It measures satisfaction (nine items) with positive personal characteristics, such as self-esteem, sense of humor, ability to relate to others, ability to show affection, and overall enjoyment of life (α total scale = 0.91).
(c) School: It measures satisfaction with the school environment (six items), including perceived importance of the school itself, the interpersonal relationships at school, and overall satisfaction with school (α total scale = 0.88).
(d) Compared self: It assesses satisfaction based on social comparisons with their peers. The six items relate to topics such as leisure activities, friendships, and fulfilling desires and affections (α total scale = 0.93).
(e) Nonviolence: It assesses the desire not to get involved in aggressive situations (six items), such as fights and arguments (α total scale = 0.80).
(f) Self-efficacy: It measures (seven items) satisfaction with the ability and competence to achieve goals. The items in this dimension relate to autonomy, leisure, material satisfaction, and fulfilling desires (α total scale = 0.85).
(g) Friendship: It assesses satisfaction with friendships (eight items), support received, and enjoyment (α total scale = 0.90).

#### 2.3.4. Statistical Analysis

Appropriate summary measures were calculated (e.g., mean, standard deviation, and coefficient of variation). Parametric bivariate tests (Student’s t-test for independent groups, Pearson’s r significance test, one-way ANOVA with Newman-Keuls post hoc procedures) were used to test hypotheses and study sample homogeneity.

Multivariate tests included canonical correlation analysis (CCA) and structural equation modeling (Marôco, 2021). IBM SPSS software (version 30) and AMOS (version 30; SPSS Inc., Chicago, IL, USA) were used in this study. Canonical correlation analysis (CCA) is used to test the relation between [Escala de Avaliação do Eu Resiliente (RSAS)] External supports, Inner strengths, Willingness to act, Social skills, Emotional well-being, Social well-being, and Psychological well-being from the Mental Health Continuum–Short Form (MHC-SF) and Family, Self, School, Compared Self, Non-violence, Self-efficacy, and Friendship, from the Multidimensional Life Satisfaction Scale for Adolescents (MLSSA).

Structural equation modeling (SEM) is used to test the relation between the global score of Mental Health Continuum–Short Form, the global score of Escala de Avaliação do Eu Resiliente and the global score of Multidimensional Life Satisfaction Scale for Adolescents.

## 3. Results

Table 1 shows the descriptive statistics for each subscale and the total scale. The coefficients of variation (CVs) reveal that WB had the highest relative dispersion of scores, with values always above 0.15. The dispersion of scores was also very high and heterogeneous in social WB (CV > 0.30). High CV values (>0.15) were also obtained in the MLSSA subscales, except for the total scale and the Friendship subscale, which both had CV values of 0.14.

Although the results were not presented in tables, it should be noted that after coding the total RSAS and MHC-SF scores in terms of resilience, 0.7% of adolescents had a low level, 18.5% had a medium level, and 80.8% had a high level. Regarding MH, 3.0% exhibited languishing, 32.1% exhibited moderate WB, and 64.0% exhibited flourishing. Kendall’s tau-b correlation coefficient revealed a positive, moderate, and statistically significant association between the categories of variables (*p* < 0.001).

Subsequently, the results of the scales were compared (Table 2). In this case, only the total scores of the scales were used to avoid excessive statistical data accumulation that would have no practical effect on the analysis, as the scores of all subscales will be used in the canonical correlation analysis (CCA).

As can be seen in the ANOVA results (including post hoc tests), all differences found in total subscale scores were statistically significant (*p* < 0.001). Taking the calculated effect size measures as a reference, the effect was low on the RSAS (*η*^2^ = 0.03) and medium on the other subscales (*η*^2^ = 0.08), namely on the total scores of the MLSSA (*η*^2^ = 0.12) and the MHC-SF, being even more significant on the latter.

Post hoc tests using the Student–Newman–Keuls method revealed differences among all groups for MHC-SF scores and between 5th-6th grades, 7th–9th grades, and 10th–12th grades for RSAS and MLSSA. On all scales, adolescents in 5th–6th grades—who are younger—had higher mean scores.

We proceeded to calculate Pearson’s correlation coefficients between age and the scores for resilience, life satisfaction, and well-being. The correlations between age and well-being (MHC) ranged from r = −0.374 (age and social wellbeing) to r= −0.244 (age and emotional wellbeing). Although all correlations achieved statistical significance, given the sample size, they were uniformly negative and demonstrated only modest strength. A similar trend was observed for the correlations between age and resilience, where all correlations were negative and weak, oscillating between r = −0.071 (age and personal strengths) and r = −0.211 (age and external support). Regarding the correlation between age and satisfaction, all values were also negative, ranging from r = −0.367 (age and school) to r = −0.072.

The overall pattern of consistently negative, very weak to modest correlations is a common and important finding in adolescent development research. It suggests the existence of a developmental pressure, and the observed decline in well-being and satisfaction may reflect the inherent burden and difficulty associated with these developmental tasks.

The next step was to perform the CCA. Set A included the scores for the MHC-SF and MLSSA dimensions. Set B included the scores for the RSAS dimensions.

As shown in Table 3, the first canonical function was the most important, indicating a strong correlation between the data sets (*r* = 0.873). The second and third canonical functions were statistically significant but less important, with *r*-values of 0.374 and 0.224, respectively. These modest *r*-values are combined with high Wilks’ lambda values.

Table 4 shows the values of the canonical loadings in subsets A and B. As previously mentioned, the most significant function was the first one (*r* = 0.873; *WL* = 0.190). All variables had high, negative loadings and contributed strongly to the function. The largest contributors from Set A (WB and satisfaction) were Psychological WB (−0.897), Self (−0.874), self-efficacy (−0.829) and Social WB (−0.811). In Set B (resilience), the largest contributors were Willingness to Act (−0.915), Inner Strengths (−0.832), and External Supports (−0.832). This result shows that resilience indicators are associated with WB. Thus, it can be concluded that adolescents with higher levels of mental WB and life satisfaction have higher levels of internal and external resilience.

The analysis of the proportion of variance explained (Table 5) revealed a significant association between Set A (WB and satisfaction) and Set B (resilience). The first canonical function was the most relevant, with a high canonical correlation of *r* = 0.873 (*p* < 0.001). It explained 59.2% of the variance in Set A and 71.5% of the variance in Set B. Cross-redundancy was 45.0% and 54.5%, respectively, which shows that a substantial proportion of the variance in each set is explained by the other.

**Figure 1:**
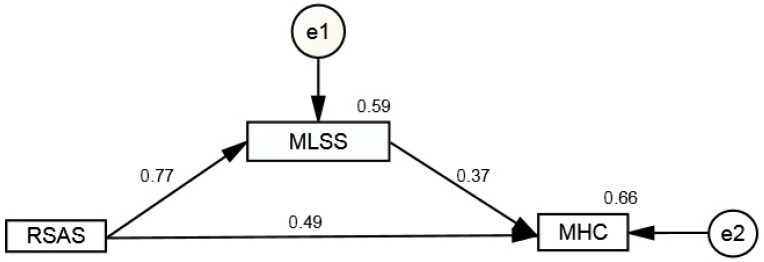
Mediation model of life satisfaction (MLSSA) on positive mental health (MHC-SF).

Finally, a mediation model of life satisfaction was tested. In this model, resilience (RSAS) was the predictor variable, life satisfaction (MLSSA) was the mediating variable, and positive MH (MHC-SF) was the dependent variable. The results are shown in Figure 1.

This model shows that all trajectories were positive and statistically significant (*p* < 0.001). WB (MHC-SF) was influenced by RSAS both directly and indirectly through MLSSA. The model shows that RSAS was a strong positive predictor of MLSSA, with a standardized regression coefficient of β = 0.77 (*p* < 0.001). MLSSA was also a significant and positive predictor of WB, with a standardized coefficient of β = 0.37 (*p* < 0.001). In addition, the analysis confirmed a direct and significant effect of RSAS on WB, with a standardized coefficient of β = 0.49 (*p* < 0.001), highlighting that the influence of RSAS on WB is not entirely mediated by MLSSA.

In the estimated model, the coefficient of determination (R^2^) for MHC-SF was 0.34, indicating that the RSAS and MLSSA variables explained approximately 34.0% of its total variance. The decomposition of effects shows that the RSAS had a significant direct impact on MHC-SF, accounting for about 24% of the explained variance. There was also a smaller indirect effect, mediated by MLSSA, accounting for around 8%. These results demonstrate that RSAS influences psychological WB directly and indirectly through MLSSA, with MLSSA partially mediating the relationship. However, the model explained only 34% of the variance in MHC-SF, suggesting the existence of other relevant factors not included in the analysis.

## 4. Discussion

The results of this study reinforce the importance of resilience, life satisfaction, and mental WB in understanding adolescence. Although adolescence is often considered the healthiest period of human life, it has been overlooked in public health (Orth & van Wyk, 2022). However, evidence suggests that adolescence is also a complex period during which MH problems manifest in unique ways. These results further our understanding of the relationship between these three variables (Azpiazu Izaguirre et al., 2021) and are essential for studying adolescent development and positive adaptation, as well as for designing interventions that promote MH and WB (Rodríguez-Fernández et al., 2016).

It is also worth mentioning that, along with WB, resilience has played a central role in systematic literature reviews (Mesman et al., 2021; Iasiello et al., 2022; Orth et al., 2022; Orth & van Wyk, 2022; Orban et al., 2024).

### 4.1. Resilience, Life Satisfaction and Well-Being Evaluation and Differences According to Educational Attainment

Summary statistics for the scales and subscales indicate that scores obtained on the WB scales, specifically social WB, are more variable, suggesting that adolescents have more diverse perceptions and subjective assessments of WB in this area. It should be noted that the negative asymmetries indicate a concentration of high scores, or a right-tail skew, in the three measures. These results are consistent with the percentages of individuals who scored at a high level of resilience (80.8%) and flourishing (64.0%). A consistent set of results emerges when comparing the three central variables (resilience, WB, and life satisfaction) across the school grades. Statistically significant differences were found in the total scores for all scales, with a linear decrease as one progresses in school.

In this case, this result may be due to a combination of the school grade they attend and their age, which is consistent with the findings reported in other studies (Goldbeck et al., 2007; Aymerich et al., 2021; Willroth et al., 2021). Older adolescents tend to have a more negative perception than younger ones. Effect size measures reinforce this trend, which is more pronounced in terms of life satisfaction and WB. However, the effect size is smaller in terms of resilience, with differences mainly resulting from sample size.

Adolescence is a time of rapid biological changes combined with new social pressures, greater academic demands, and increased interpersonal demands. Therefore, it is reasonable to consider that the sum or interconnection of these factors may contribute to a decrease in WB, even if it recovers at a later age.

### 4.2. Variable Relations and Causal Model

Regarding CCA, the data revealed a strong correlation between resilience and mental WB/life satisfaction (*r* = 0.873), which suggests that adolescents with a greater capacity for action and both internal and external support tend to exhibit higher levels of WB across its three components, including life satisfaction.

This finding is significant because it demonstrates that resilience is a multifaceted resource associated with perceptions of control, social integration, and emotional balance. These results are supported by studies showing that higher resilience in adolescents is associated with greater WB and fewer MH issues (Mesman et al., 2021).

At the same time, resilience is a multifaceted and multisystemic factor, in line with the review on the concept of resilience as a dynamic process (Mesman et al., 2021).

With regard to the latest analysis, we must first seek to explain, in comparative terms, its contribution to the CCA and why it was carried out subsequently. The CCA measures the overall association between two sets of variables, whereas mediation analysis (MA) introduces a causal hypothesis between the variables, seeking to explain the “how” and “why” of the results found in the CCA. In the latter case, composite variables (canonical functions) were used, and MA analyzed direct effects (RSAS → MHC-SF) and indirect effects (RSAS → MLSSA → MHC-SF).

This analysis corroborated the results of the CCA, which examined the nature of the relationships and showed that resilience influences WB directly and indirectly through life satisfaction, which acts as a partial mediator. In general, these results provide evidence that resilience influences WB directly and indirectly, which shows that life satisfaction is a relevant mechanism through which resilience promotes mental WB. The partial mediation also indicates that resilience continues to impact mental WB directly and independently.

The findings of this study are consistent with existing evidence (Vivas-Fernandez et al., 2024; Xu et al., 2025), which shows that resilience plays a central role in promoting mental WB (Usán Supervía et al., 2022; Marquez et al., 2023; Carvalho et al., 2024; Zhu et al., 2025).

### 4.3. Limitations

One limitation of this study is its cross-sectional design, which prevents us from making causal inferences. However, the overall analysis yielded statistical evidence.

Despite the presence of researchers during all data collection sessions, the participants’ written self-reports may still be subject to social desirability bias. Consequently, despite the large sample size, bias is always associated with this data collection approach.

As previously mentioned, although the sample size is large, the adolescents were only recruited from schools in a municipality with distinct suburban characteristics. Therefore, it is uncertain whether these adolescents are representative of all Portuguese adolescents. Future studies should therefore consider broadening the sample to include other municipalities and schools in Portugal to strengthen the generalizability of the results.

## 5. Conclusions

This study provides additional and consistent evidence on the importance of resilience, life satisfaction, and mental WB in the lives of adolescents, which are fundamental to understanding positive development during adolescence.

High levels of resilience and positive MH confirm the adaptive and growth potential of these individuals. However, variability measures in some dimensions, such as social WB, suggest a heterogeneity of subjective experiences. This implies the need for tailored, differentiated, and culturally sensitive measures to promote MH and WB.

The decrease observed on all scales as students progress through their studies suggests a less positive perception of themselves and their context, which may be related to age. These results are consistent with the available literature, indicating a decline in mental WB and life satisfaction during middle and late adolescence. This decline may result from academic demands, social pressures, and identity crises typical of this stage of human development.

The robust relationship revealed by CCA reinforces the role of resilience as a cross-cutting factor linked to both the perception of personal control and emotional balance, confirming the importance of resilience as a protective mechanism in adolescent development.

MA suggests that life satisfaction is an important mechanism in the relationship between resilience and WB, highlighting the need to enhance contexts that increase adolescents’ life satisfaction. The results emphasize the importance of creating programs that develop skills and promote positive experiences to reinforce life satisfaction and increase WB among adolescents.

Future research should prioritize longitudinal designs that allow for the analysis of causal relationships and the inclusion of additional variables.

## Figures and Tables

**Table 1:** Summary statistics of the subscales and total scales (N = 589).

Scales	Min.	Max.	M	SD	Md	CV	SK	Ku
External supports	8.00	24.00	17.58	2.42	18.00	0.14	−1.15	1.59
Inner strengths	4.00	18.00	13.24	1.72	14.00	0.13	−1.20	2.45
Willingness to act	3.00	12.00	8.23	1.38	8.00	0.17	−0.67	0.26
Social skills	6.00	30.00	20.20	3.74	21.00	0.18	−0.81	0.82
Total of RSAS	23.00	84.00	59.12	7.99	61.00	0.14	−0.98	1.79
Emotional well-being	0.00	5.00	3.95	0.93	4.00	0.23	1.30	165
Social well-being	0.00	5.00	3.25	1.22	3.60	0.38	−0.73	−0.40
Psychological well-being	0.17	5.00	3.71	1.01	4.00	0.27	−1.13	1.08
Total of MHC-SF	0.29	5.00	3.60	0.99	3.86	0.27	−0.93	0.39
Family	10.00	50.00	43.58	7.25	46.00	0.17	−1.55	2.34
Self	9.00	45.00	34.49	7.58	36.00	0.22	−0.85	0.22
School	6.00	30.00	22.74	4.68	23.00	0.21	−0.68	0.44
Compared Self	6.00	30.00	20.57	5.30	20.00	0.26	−0.16	−0.50
Non-violence	11.00	30.00	23.19	3.89	23.00	0.17	−0.48	0.03
Self-efficacy	7.00	35.00	25.93	4.42	26.00	0.17	−0.66	0.92
Friendship	10.00	40.00	34.01	4.81	35.00	0.14	−1.45	3.13
Total of MLSSA	78.00	258.00	205.34	29.15	205.39	0.14	−0.87	0.80

Min. = Minimum; Max. = Maximum; M = mean; SD = Standard deviation; Md = Median; CV = Coefficient of variation; SK = Skewness.; Ku = Kurtosis.

**Table 2:** Results of the one-way ANOVA test on the total scale scores, according to the adolescents’ educational attainment.

Total Scores	5th–6th Grades		7th–9th Grades		10th–12th Grades		F	*η* ^2^	Post Hoc Tests ^§^		
	M	SD	M	SD	M	SD			ab	ac	bc
MHC-SF	4.15 ^a^	0.74	3.57 ^b^	0.98	3.20 ^c^	0.97	38.685 ***	0.12	*	*	*
RSAS	61.63 ^a^	6.60	58.74 ^b^	8.55	57.68 ^c^	7.66	10.287 ***	0.03	*	*	^ns^
MLSSA	219.25 ^a^	22.95	203.28 ^b^	29.46	197.86 ^c^	29.45	20.174 ***	0.08	*	*	^ns^

M = Mean; SD = Standard deviation; *** = *p*< 0.001; * = *p*< 0.05; ^§^ = Student-Newman-Keuls test. a = 5th–6th Grades; b = 7th–9th Grades and c = 10th–12th Grades.

**Table 3:** Canonical correlations (N = 589).

Canonical Function	r	Eigenvalue	WL	F	*p*
1	0.873	3.190	0.190	24.193	0.000
2	0.374	0.163	0.796	4.082	0.000
3	0.224	0.053	0.926	2.277	0.003
4	0.158	0.025	0.975	1.699	0.107

WL = Wilks’ lambda.

**Table 4:** Canonical loadings for Sets A and B.

Variables	1	2	3	4
Set A				
Emotional well-being	−0.806	−0.052	0.082	−0.273
Social well-being	−0.811	0.053	−0.192	−0.283
Psychological well-being	−0.897	−0.033	0.160	−0.210
Family	−0.806	0.276	0.197	0.293
Self	−0.874	−0.278	−0.101	0.062
School	−0.726	0.157	−0.170	−0.045
Compared Self	−0.472	0.098	0.185	0.376
Non-violence	−0.570	−0.397	−0.189	0.435
Self-efficacy	−0.829	−0.147	0.115	−0.223
Friendship	−0.794	0.281	−0.364	0.063
Set B	−0.800	0.584	0.019	−0.134
External supports	−0.832	0.154	−0.274	0.456
Inner strengths	−0.832	−0.336	−0.324	−0.300
Willingness to act	−0.915	−0.151	0.372	0.053
Social skills	−0.800	0.584	0.019	−0.134

**Table 5:** Proportion of Variance Explained.

Canonical Variable	Set A by Self	Set A by Set B	Set B by Self	Set B by Set A
1	0.592	0.450	0.715	0.545
2	0.045	0.006	0.125	0.018
3	0.036	0.002	0.080	0.004
4	0.067	0.002	0.080	0.002

## Data Availability

The datasets generated and analyzed during the current study are available from the corresponding author upon reasonable request. Requests will be reviewed and granted in compliance with ethical and legal considerations.

## Abbreviations

The following abbreviations are used in this manuscript:

CCA	Canonical Correlation Analysis
CV	Coefficient of variation
HBSC	Health Behavior in School-aged Children
M	Mean
MA	Mediation analyses
Max.	Maximum
Md	Median
MH	Mental Health
MHC-SF	Mental Health Continuum–Short Form
Min.	Minimum
MLSSA	Multidimensional Life Satisfaction Scale for Adolescents
RSAS	Resilient Self-Assessment Scale
SD	Standard deviation
SK	Skewness
WB	Well-being
WHO	World Health Organization
WL	Wilks’ lambda
